# [Retracted] miR‑613 suppresses migration and invasion in esophageal squamous cell carcinoma via the targeting of G6PD

**DOI:** 10.3892/etm.2023.12246

**Published:** 2023-10-10

**Authors:** Xiangyu Su, Chanchan Gao, Xiaoyao Feng, Ming Jiang

Exp Ther Med 19:3081–3089, 2020; DOI: 10.3892/etm.2020.8540

Following the publication of this paper, it was drawn to the Editors’ attention by a concerned reader that the Transwell invasion assay data shown in Fig. 6B on p. 3085 were strikingly similar to data appearing in different form in other articles written by different authors at different research institutes that had either already been published elsewhere prior to the submission of this paper to *Experimental and Therapeutic Medicine*, or were under consideration for publication at around the same time. Moreover, three overlapping data panels were identified in Fig. 6A, such that data which were intended to show the results from differently performed experiments appeared to have been derived from the same original source(s). In view of the fact that certain of these data had already apparently been published previously, and given the lack of rigour on the part of the authors in assembling the figures, the Editor of *Experimental and Therapeutic Medicine* has decided that this paper should be retracted from the Journal. After having been in contact with the authors, they agreed with the decision to retract the paper. The Editor apologizes to the readership for any inconvenience caused.

